# Audit on Compliance to Guidelines in CT Scanning for Urolithiasis

**DOI:** 10.5334/jbsr.2941

**Published:** 2022-12-09

**Authors:** Blandine Defrenne, Nigel Howarth, Denis Tack

**Affiliations:** 1 Université Libre de Bruxelles, Faculté de Médecine, Route de Lennik 808, B-1070 Bruxelles, BE; 2 Clinique des Grangettes, 7, chemin des Grangettes, CH- 1224 – Chêne-Bougeries, CH; 3 Epicura Hospital, Rue Louis Caty 136, B-7331 Baudour, BE

**Keywords:** computed tomography, quality control, radiation dose, urolithiasis, audit

## Abstract

**Introduction:**

According to the ALARA principle, CT-imaging procedures should be implemented to optimize radiation doses. The purpose of this study is to determine whether a quality control process has an impact on compliance with procedures.

**Materials and methods:**

This retrospective study was conducted in three hospitals, focusing on the selection of the appropriate acquisition protocol and the reduction of acquisition height in abdominal computed tomography (CT) examinations performed to diagnose or rule out urolithiasis. A first audit was conducted to measure the compliance with the procedure. Next, a reminder of the CT-urolithiasis procedure was given to stakeholders. Three months later, a second audit was conducted to measure the impact of the repeat recall information on compliance, and to compare the outcome with an earlier audit conducted five years earlier.

**Results:**

We included 517 ‘urolithiasis CT examinations’. The compliance ranged from 41.67% to 64.8% for the first audit. After the reminder of the urolithiasis procedure, compliance ranged from 50% to 76.10%. This improvement was statistically significant for hospital A and B (p < 0.001 for hospital A, p = 0.013 for hospital B) but not for hospital C (p = 0.405). Despite prior demonstration that improved compliance persisted at one year from an initial audit, our actual data show that this compliance had decreased at year five, confirming the need to repeat compliance audits more frequently, or to monitor it continuously.

**Conclusion:**

Surveying compliance to procedures can improve compliance but only for a limited duration. Monitoring compliance more frequently or even continuously is recommended.

## 1. Introduction

Over the last decades, the quality of diagnostic imaging studies has improved significantly, and the number of studies increased for both irradiating and non-irradiating techniques.

In Belgium, collective radiation due to medical imaging increased until 2010, before reversing since 2015, thanks to both optimization of radiology examinations and modernization of radiology equipment [1]. Increase in collective radiation dose can increase the stochastic risk and potentially induce cancers [2]. Limiting/reducing this collective dose delivered to patients is important and has been addressed in two consecutive European Directives (97/43, 2013/59). Two concepts derive from the radioprotection concept: justification and optimization. Justification is a benefit-risk balance leaning towards benefit while optimization involves taking the lowest possible dose that is sufficient to generate an image of adequate quality for diagnosis. To guarantee compliance with these two concepts, procedures are established in hospitals. Compliance with procedures, however, is largely operator dependent [3].

Urolithiasis is a highly prevalent condition with a high recurrence rate which can reach 50% at five years [4]. Patients suffering from renal calculi are subject to multiple computed tomography (CT) examinations during their lifetime and sometimes from a young age. Reducing their cumulative radiation dose is an important issue [5].

Dose reduction techniques include reduction in tube current with subsequent increased image noise, and reduction in acquisition length compared with a standard abdominal CT examination, typically by using the root of the diaphragm as apical landmark instead of top of the liver. Low-dose protocols allow to reduce the irradiation dose without compromising the detection of many diseases, including alternative diagnoses [6,7,8]. They have already proven their value for the detection of urolithiasis and are an integral part of current recommendations [8,9].

In our department, a dedicate low-dose CT-acquisition procedure in patients with suspected urolithiasis has been introduced since 2005. One could hypothesize that 1) surveying compliance with this procedure and 2) informing the radiology staff on the compliance, could improve compliance over time.

The aim of this study was therefore to monitor compliance to the procedure in two distinct steps: a baseline observation before reminding the radiology staff of the procedure, and an assessment after renewed attention, and to compare it with the observation obtained five years earlier [10].

## 2. Materials and Methods

According to the EU legislation (i.e., the Directive 95/46/EC), a purely observational study with complete anonymization of the data at the source, removing any possibility of identifying the individual patients, is not subject to ethical review [11].

This study was conducted in the radiology department of the EPICURA hospital, Belgium, comprising three hospital sites. Two hospital sites are equipped with the same CT device, a 64 detector-row scanner (Definition AS+, Siemens Medical Solutions, Germany), installed in 2011 and upgraded with new detectors (Stellar Detectors) in 2017. One hospital site is equipped with a more recent scanner device with 2x64 detector-rows (CT Drive, Siemens, Germany). The fourth scanner installed is a 16 detector-row device (Somatom Go Up, Siemens, Germany).

Most radiologists work in the three hospitals and the Department of Radiology has a single head for the three sites. The radiographers form three distinct teams working either in hospital A, B or C.

CT examinations from the three hospitals and their corresponding requests are archived in one and the same Picture Archiving and Communicating System (PACS) (Telemis, Belgium).

To investigate the ideal audit repetition interval, we compared our data with those of Oliveri et al. whose paper focused on hospitals B and C in the same hospital group [10]. Since then, the institution has gone through changes with both a fusion and a reorganization of the department.

### CT Procedures

When a patient is referred for a CT for suspected urolithiasis, the radiographers must follow corresponding standardized procedure to optimize the radiation dose.

Dose reduction is obtained differently according to the CT device. With definition scanner devices, the reduced dose is obtained through a reduction of the tube current-time product expressed in mAs. Compared with a standard abdominal CT protocol, the reduced dose protocol delivers 33% lower tube current-time product, and its name includes a specific low-dose label (‘LD’). The two remaining CT devices are equipped with a Tin (Sn) filter, enabling a dose reduction of more than 65% compared with the standard abdominal CT protocol. The Tin filter protocol name includes a specific label named ‘Sn’.

### Patients and Audit

CT examinations were selected through a DACS (Data Acquisition Computerized System) program containing anonymized dose reports and text of CT reports issued by radiologists. This program provided access to examinations dealing with stone diseases through several key words (‘renal colic’, ‘ureteric stone’, ‘migration’). These examinations were transferred to a dedicated server and data analysis was performed on this server, with complete anonymization of patient’s data except for their age and gender.

Information regarding CT examinations was collected retrospectively and included: patient’s age, examination indication (lithiasis control, renal colic, pain in the right or left iliac fossa), procedure choice, acquisition length, radiation dose descriptors and the radiologist’s name, which was also anonymized.

A first audit period took place from February to April 2021. During May 2021, the radiologist responsible for quality control and radioprotection in the department reminded the stakeholders of the urolithiasis procedure. A second audit analyzed data from June to August 2021.

### Image Analysis

Compliance with the procedure was evaluated according to two criteria: the selection of the appropriate acquisition protocol and the reduction of acquisition height with exclusion of the top of the liver. For each exam, we collected the DLP and CTDI_volume_.

### Statistical Analysis

We calculated the proportion of examinations performed according to the urolithiasis procedure amongst all the urolithiasis CT scans performed during both audit periods and per radiologist. The proportion comparisons were calculated using the χ^2^ Pearson test.

We performed a Shapiro-Wilk test to testify the normality of our quantitative variables.

We compared medians using the Mann-Whitney test. Statistical significance was set for p values < 0.05.

## 3. Results

We included 517 urolithiasis CT examinations. The median patient age was 50 years for the first period and 50 years for the second period (p = 0.770).

### Urolithiasis Procedure

Table 1 summarizes the percentages and proportions of urolithiasis examinations conducted in compliance with the procedure for the three hospitals for both periods.

**Table 1:** Compliance with Urolithiasis Procedure according to Hospitals.


		FIRST AUDIT	SECOND AUDIT	P VALUE

**Hospital A**	0 criterion	35.2% (44/125)	23.9% (38/159)	<0.001

	1 criterion	52.8% (66/125)	38.4% (61/159)	

	2 criteria	12% (15/125)	37.7% (60/159)	

	At least 1 criterion	64.8% (81/125)	76.10% (121/159)	0.037

**Hospital B**	0 criterion	53.9% (55/102)	38.9% (44/113)	0.013

	1 criterion	29.4% (30/102)	27.5% (31/113)	

	2 criteria	16.7% (17/102)	33.6% (38/113)	

	At least 1 criterion	46.08% (47/102)	61.06% (69/113)	0.028

**Hospital C**	0 criterion	58.33% (7/12)	50% (3/6)	0.501

	1 criterion	25% (3/12)	50% (3/6)	

	2 criteria	16.67% (2/12)	0% (0/6)	

	At least 1 criterion	41.67% (5/12)	50% (3/6)	0.740

**Hospital A. B and C**	0 criterion	44.35% (106/239)	30.94% (86/278)	<0.001

	1 criterion	41.42% (99/239)	34.17% (95/278)	

	2 criteria	14.23% (34/239)	35.25% (98/278)	

	At least 1 criterion	55.65% (133/239)	69.42% (193/278)	0.00117


We reached statistical significance for hospital A and B (p < 0.001 and p = 0.013 respectively) but not for hospital C (p = 0.405).

**Figure 1:**
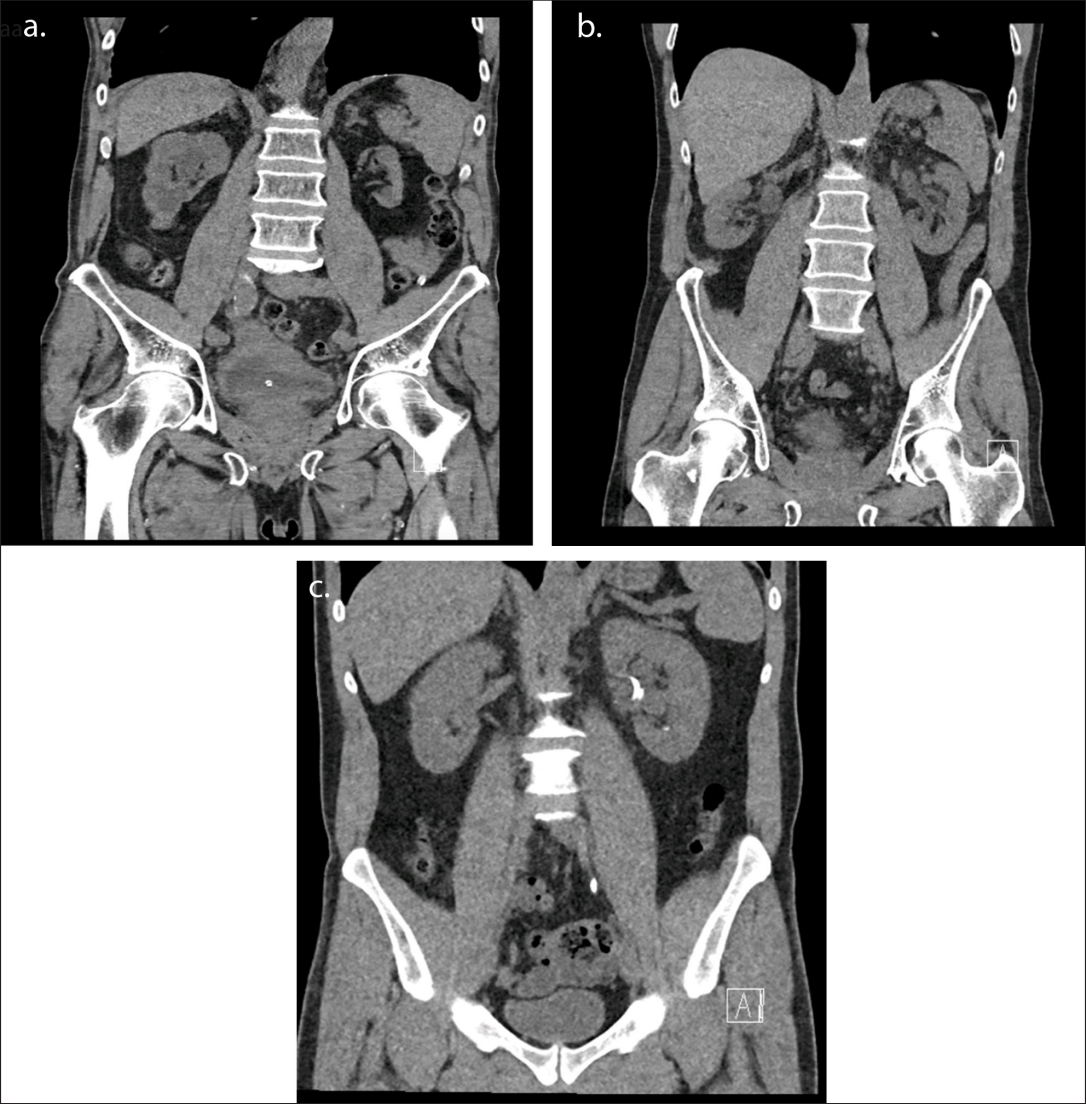
CT examination for urolithiasis in coronal section for three patients of similar diameters: **(a)** wrong acquisition length and protocol selection – DLP 206.1 mGy.cm **(b)** right protocol but wrong acquisition length – DLP 82.4 mGy.cm **(c)** right protocol selection using Tin Filter and right acquisition length – DLP 34.3 mGy.cm.

Figure 1 illustrates 3 examples of CT-scans in the coronal plane from patients with similar diameters and different acquisition lengths and/or protocol selections.

### Radiologist Dependency

Table 2 summarizes the percentages and proportions of urolithiasis examinations conducted in compliance with the procedure for each radiologist.

**Table 2:** Compliance with Urolithiasis Procedure according to Radiologists.


		FIRST AUDIT	SECOND AUDIT

**RAD1**	0 criterion	41.18% (7/17)	34.21% (13/38)

	At least 1 criterion	58.82% (10/17)	65.79% (25/38)

**RAD2**	0 criterion	45% (9/20)	24.14% (7/29)

	At least 1 criterion	55% (11/20)	75.86% (22/29)

**RAD3**	0 criterion	55.56% (10/18)	33.33% (8/24)

	At least 1 criterion	44.44% (8/18)	66.67% (16/24)

**RAD4**	0 criterion	54.55% (6/11)	56.25% (9/16)

	At least 1 criterion	45.45% (5/11)	43.75% (7/16)

**RAD5**	0 criterion	62.5% (10/16)	28.57% (4/14)

	At least 1 criterion	37.5% (6/16)	71.43% (10/14)

**RAD6**	0 criterion	55.56% (10/18)	23.08% (3/13)

	At least 1 criterion	44.44% (8/18)	76.92% (10/13)

**RAD7**	0 criterion	0% (0/7)	11.11% (1/9)

	At least 1 criterion	100% (7/7)	88.89% (8/9)

**RAD8**	0 criterion	43.75% (7/16)	40% (6/15)

	At least 1 criterion	56.25% (9/16)	60% (9/15)

**RAD9**	0 criterion	25.81% (8/31)	16.67% (5/30)

	At least 1 criterion	74.19% (23/31)	83.33% (25/30)

**RAD10**	0 criterion	45% (9/20)	14.29% (2/14)

	At least 1 criterion	55% (11/20)	85.71% (12/14)

**RAD11**	0 criterion	0% (0/3)	11.11% (2/18)

	At least 1 criterion	100% (3/3)	88.89% (16/18)

**RAD12**	0 criterion	60% (6/10)	100% (2/2)

	At least 1 criterion	40% (4/10)	0% (0/2)


*Note*: RAD = Radiologist.

### Radiation Dose

The median DLP was 162.6 mGy.cm (p25: 77 | p75: 256.2) for the first audit and 95.2 mGy.cm (p25: 58.65 | p75: 216) for the second audit (p < 0.001).

### Audit Interval

Compared with Oliveri’s one-year control, which had reached a compliance of 85%, our first audit conducted five years later shows a drop to 55.65% of the compliance to the urolithiasis procedure and our second audit reached 69.42%.

## 4. Discussion

Our study showed that 1) while procedures are established to optimize the radiation dose, compliance remains imperfect; 2) surveying compliance with procedures and informing the radiology staff on this compliance increases compliance over time; 3) the increase is not statistically significant for every hospital, even though they belong to the same health group and radiology department; 4) compliance with the second criteria (shortening the acquisition length) is lower than that of selecting the appropriate reduced-dose protocol; and 5) compared with the 2016 study, compliance dropped.

### These observations deserve further discussion.

First, regarding the impact of surveying compliance, our findings are consistent with those reported by Oliveri et al. showing an increased compliance after informing the staff [10]. However, they also demonstrate that the compliance which proved to be stable at one year, decreased thereafter.

Indeed, the percentage of compliance with the urolithiasis procedure had reached 85% by the end of the study, but our first audit, which took place in the same radiology department five years later, reached only 55.65%. This suggests that the effect of one quality control process on compliance decreases over time. To ensure a sustainable improvement with compliance, these audits should be repeated regularly, as required by the Federal Agency for Nuclear Control (FANC) since 2020 [12], or even better, monitored continuously using DACS or similar applications. The optimal delay for repeating the audit is not yet known and would probably depend on several human and organizational factors specific to each department.

Second, even if compliance increased, it does not reach perfection. Indeed, our audit showed an increase in compliance of 15% and reduction of the median DLP by 41.45% (p < 0.001). The reduction achieved in DLP shows that, even if it seems low, it has a real impact on the radiation dose delivered to the patient. However, the DLP can be influenced by the patient diameter, a value which we unfortunately did not have at our disposal.

As illustrated in Figure 1, the dose reduction can be very important, as high as 83%, which should largely motivate stakeholders to use low-dose protocols.

However, even if not perfect, compared with data from the literature, the use of reduced-dose CT in urolithiasis in our department is much higher than reported elsewhere [13]. Indeed, Weisenthal et al. reported that the use of this protocol is infrequent and as low as 5.6%. The corresponding DLP was 689 mGy.cm, seven times higher than in our study.

Thirdly, we found that radiologists showed varying degrees of compliance ranging from 37.5% to 100% for the first audit and from 0% to 88.89% for the second audit.

Other studies have already shown radiologist’s dependence for adhesion to guidelines and found factors associated with a higher rate of compliance, such as practice in a teaching hospital setting or fewer than five years of experience [14]. Another reason that could explain such differences could be a fear of inadequate image quality, even though studies have shown the same diagnostic performance with low-dose CT [6,7,8].

Fourthly, compliance with the first criterion (low-dose protocol) is higher than compliance with the second (acquisition length). This could be because low-dose protocol selection requires less interaction on the scanner console than lowering the acquisition length. Some radiologists may also be reluctant to lower the acquisition length by fear of missing an alternative diagnosis, despite the evidence by Brassard et al. that alternative diagnosis are detectable on the restricted abdominal coverage [15].

Lastly, we reached statistical significance for hospital A and B but not for hospital C. This could be because hospital C conducted less scans for urolithiasis during the same period. This has been found to be a significant factor in other articles, such as one by Eisenberg et al., who showed that adhesion to Fleischner guidelines in chest imaging was dependent on the number of CT scans for small nodule detection in each center [14].

One limitation of our study is that we only conducted a single-component intervention. A study by Smith-Bindman showed that detailed feedback on CT radiation dose along with actionable dose-lowering suggestions reduced doses more significantly than an audit feedback alone [16]. We could train radiographers continuously by setting this topic on a regular basis to be discussed in meeting with the staff.

Another limitation is that we were unable to investigate the potential impact of the time of day that the examinations were performed. Is there an impact of night duty or of the number of radiographers present in the department? More data will be available soon, thanks to new functionalities of the DACS.

## 5. Conclusion

In conclusion, the results of this study suggest that even if surveying compliance with appropriate procedures and informing the radiology staff increases it over time, compliance will drop gradually if not surveyed on a regular basis or monitored continuously.
